# Immunoglobulin Superfamily Virus Receptors and the Evolution of Adaptive Immunity

**DOI:** 10.1371/journal.ppat.1000481

**Published:** 2009-11-26

**Authors:** Terence S. Dermody, Eva Kirchner, Kristen M. Guglielmi, Thilo Stehle

**Affiliations:** 1 Department of Pediatrics, Vanderbilt University School of Medicine, Nashville, Tennessee, United States of America; 2 Department of Microbiology and Immunology, Vanderbilt University School of Medicine, Nashville, Tennessee, United States of America; 3 Elizabeth B. Lamb Center for Pediatric Research, Vanderbilt University School of Medicine, Nashville, Tennessee, United States of America; 4 Interfaculty Institute for Biochemistry, University of Tuebingen, Tuebingen, Germany; The Scripps Research Institute, United States of America

Obligate intracellular pathogens depend on cell-surface molecules to attach and enter into host cells. Pathogen receptors may be highly specialized proteins, such as complement receptors or neurotransmitter receptors, or more ubiquitous components of cell membranes, such as integrins or sialic acid–containing oligosaccharides. The immunoglobulin superfamily (IgSF) of molecules contains several members that are expressed at the cell surface, bind diverse ligands, and contribute to a variety of cellular activities, including adhesion and immune responses. Many viruses have usurped the adhesive properties of IgSF proteins to mediate attachment (Table 1). Strategies used by viruses to engage IgSF receptors provide clues to general mechanisms by which IgSF proteins bind different types of ligands, including antigens.

**Table 1:** IgSF Receptors Used by Selected Viruses.

Virus	Receptor	Number of Immunoglobulin Domains	References
Adenovirus	Coxsackievirus and adenovirus receptor (CAR)	2	[31],[32]
Coronavirus	Carcinoembryonic antigen glycoprotein family (CEACAM)	4	[33]–[35]
Coxsackievirus B	Coxsackievirus and adenovirus receptor (CAR)	2	[31],[32]
Herpes simplex virus	Nectin-1 (PRR1/HveC)	3	[36]
	Nectin-2 (PRR2/HveB)	3	[37]
Human immunodeficiency virus	CD4	4	[38],[39]
Measles virus	Signaling lymphocyte-activation molecule (SLAM)	2	[40]
Poliovirus	Poliovirus receptor (PVR, CD155)	3	[41]
Rabies virus	Neural cell adhesion molecule (NCAM-1, CD56)	5	[42]
Reovirus	Junctional adhesion molecule-A (JAM-A)	2	[28],[43]
Rhinovirus	Intercellular adhesion molecule-1 (ICAM-1)	5	[44]–[46]

Members of the IgSF have diverged in sequence and function. However, all contain domains with the characteristic immunoglobulin fold, which is defined by two opposing antiparallel β-sheets connected in a unique manner [1],[2]. The core of the immunoglobulin fold is formed by four β-strands (B, C, E, and F) augmented with three to five additional β-strands (A, C′, C″, D, and G) to yield several distinct subtypes [1],[2]. Most common are the V-set and C-set immunoglobulin domains, which are named according to their occurrence in the variable and constant regions of immunoglobulins, respectively. A third type, the I-set, is an intermediate structure between the V- and C-sets found frequently in cell-surface receptors. Immunoglobulin domains rarely occur in isolation but typically form concatenated chains, often with a V-set or I-set domain at the N-terminus.

Biochemical and structural analyses of interactions between viruses and their cognate IgSF receptors reveal several striking similarities. First, in cases in which structural information about virus–receptor complexes is available, the viral attachment proteins exclusively bind to the most membrane-distal, N-terminal domain (D1) of the IgSF receptors [3]–[10]. While structural information about complex formation is lacking for the IgSF receptors carcinoembryonic antigen-related cell adhesion molecule, nectin-1, nectin-2, and signaling lymphocyte-activation molecule (SLAM), biochemical studies also implicate their respective D1 domains in virus binding [11]–[14]. Second, virus-contacting residues lie towards the upper “tip” of the IgSF D1 domain. Third, the viral receptor-binding region engages the CC′FG β-sheet of the IgSF receptor D1 domain. Fourth and finally, almost all of the receptor domains interacting with viruses belong to the V-type IgSF fold. The single exception, the D1 domain of ICAM-1, belongs to the I-set type, which is structurally similar to the V-set domain.

**Figure 1:**
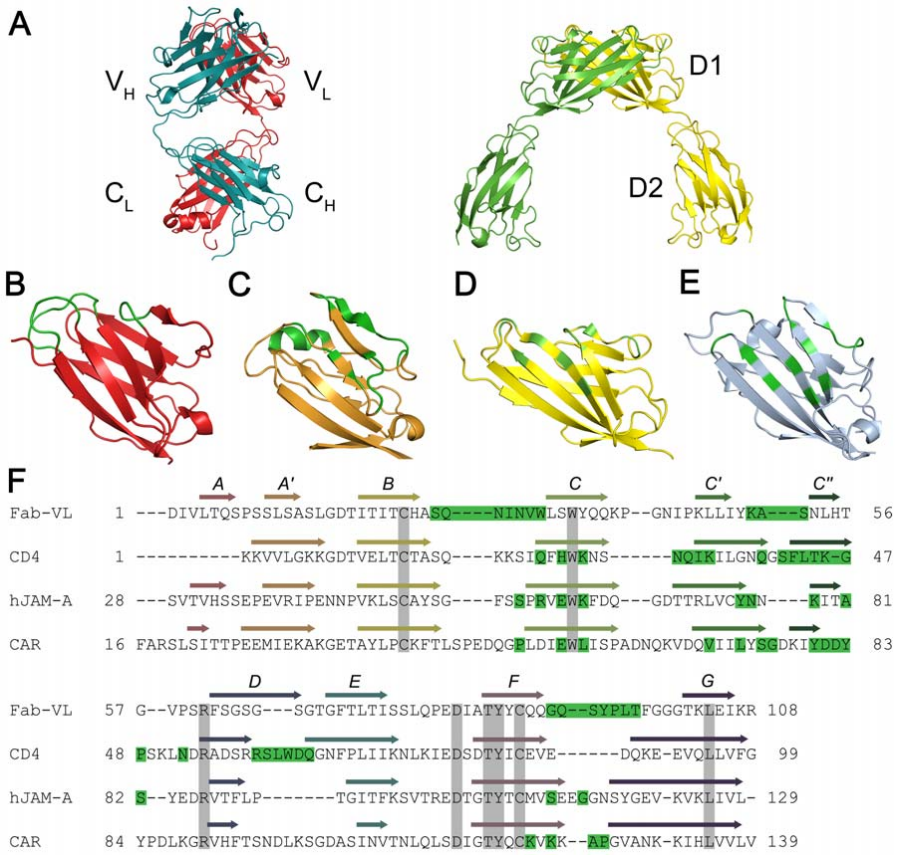
Contact areas in Fab and virus receptors. (A) Ribbon drawing of mFab 231 (left) ([27]; 1IGT) and the extracellular domains of hJAM-A (right) ([28]; 1NBQ). Variable (V) and constant (C) domains of heavy (H) and light (L) chains and D1 and D2 domains of JAM-A are labeled. (B) Ribbon drawing of the variable domain of the light chain (V_L_) of the mFab shown in (A). CDRs are colored green. (C–E) Ribbon drawings of the complexed D1 domains of (C) CD4 ([4]; 1GC1), (D) hJAM-A ([9]; 3EOY), and (E) CAR ([5]; 1KAC). Residues contacting the virus proteins with a distance cutoff of 4 Å are colored green. (F) Structural alignment of mFab 231 V_L_ ([27]; 1IGT), CD4 D1 ([29]; 1CDJ), hJAM-A D1 ([28]; 1NBQ), and CAR D1 ([30]; 1EAJ) performed using MODELLER (program Web site: http://salilab.org/modeller/). β-strands are indicated, and conserved residues are highlighted in grey. mFab 231 V_L_ CDRs and residues in CD4, hJAM-A, and CAR that contact the viral attachment proteins gp120, σ1, and fiber, respectively, with a distance cutoff of 4 Å, are highlighted in green.

Although the database of viral proteins in complex with IgSF receptors is still quite small, interactions of viruses with their receptors parallel the recognition mode of immunoglobulins, which also recognize their cognate antigens via residues at the tip of their N-terminal, V-set domains. The case of the receptor-binding head domain of reovirus attachment protein σ1 in complex with the D1 domain of its receptor, junctional adhesion molecule-A (JAM-A) [9], serves to illustrate this point (Figure 1A). The JAM-A homodimer strikingly resembles the dimer formed by the V-set domains of the light and heavy chains of immunoglobulins. In both structures, the two V-set domains face each other with similar orientations. Moreover, residues in the receptor required for virus attachment reside in β-strands and intervening loops that juxtapose the complementarity determining regions (CDRs) of antibody molecules. Thus, residues known to interact with ligands map to corresponding regions near the tip and one side of the V-set domains. These similarities extend beyond reovirus receptor JAM-A. Other IgSF virus receptors, such as the coxsackievirus and adenovirus receptor (CAR) [5] and HIV receptor CD4 [4], also recognize their viral ligands via residues that partially overlap with the CDR region of immunoglobulins (Figure 1B–F). CAR forms a homodimer via its D1 domain that is very similar to the JAM-A homodimer [15]. CD4 also forms homodimers, albeit via its D4 domain [16].

The immunoglobulin fold predates the evolution of vertebrates. Genomes of invertebrate organisms encode numerous molecules that belong to two families with homologs in vertebrates: the JAM/cortical thymocyte marker of *Xenopus* (CTX) family and the nectin family [17]. Vertebrate counterparts of these genes are found in discrete blocks, and many are now diversified to encode molecules that function in adaptive immunity, including CD3 and SLAM [17]. Invertebrates do not encode recombination-activating genes (RAGs) and generally display only limited antigen-specific immunity. Therefore, the core structural element of adaptive immunity, the immunoglobulin fold, evolved prior to a mechanism to generate a highly diversified antigen-specific repertoire.

Similarities in mechanisms of ligand engagement by IgSF pathogen receptors and immunoglobulins, coupled with the evolution of the immunoglobulin fold prior to the existence of the vertebrate adaptive immune system, suggest the possibility that primitive members of the JAM/CTX and nectin families evolved to become soluble adaptive immune mediators in modern vertebrates. One attractive hypothesis is that soluble forms of pathogen receptors served as precursors to molecules of the adaptive immune system. Soluble receptors would neutralize viral infection by competing with surface-expressed versions of the receptor for binding sites on the virus. In modern vertebrates, some viruses manipulate surface-expressed and soluble forms of their receptors to maximize the efficiency of infection. For example, human rhinovirus upregulates membrane-bound ICAM-1, while diminishing expression of the soluble form of the receptor to increase target cell infectivity [18]. Expression of a soluble pathogen receptor followed by duplication within the primitive genome and acquisition of mutations that permitted recognition of additional pathogens could confer a strong selective advantage. Upon introduction of RAGs into the vertebrate genome, such a gene family would have been primed to express molecules akin to present-day immunoglobulins. Alternatively, membrane-anchored forms of IgSF molecules that arose in primitive invertebrates may have been maintained in the genome due to their cell-adhesion functions, followed by the serendipitous introduction of mechanisms for the secretion and generation of diversity. In this scenario, pathogens may have contributed to the evolution of the modern adaptive immune system at much later evolutionary times.

Is there evidence that favors either of these potential evolutionary mechanisms? In addition to similarities in their ligand-binding strategies, many of the closest structural homologs of JAM-A are immunoglobulins, which raises the possibility that immunoglobulins are more closely related to JAM-A than to other IgSF molecules. A search for structural homologs of the JAM-A D1 domain using the Dali algorithm [19] provides support for this hypothesis. The closest structural homologs of the JAM-A D1 domain are immunoglobulin domains, with the highest Dali Z-score of 14.6 for an IgAκ variable domain (PDB code 2FBJ) (Table 2). Other IgSF proteins with similarity to JAM-A D1 have significantly lower Z-scores. The Z-scores correlate well with root mean square deviations for superpositions of JAM-A D1 with immunoglobulins, which also are lower (i.e., more similar) than the corresponding values for superpositions of JAM-A D1 with other IgSF proteins. This homology search can be extended to CAR, neural cell adhesion molecule, and nectin-like molecule 1, which result in Z-scores that are generally higher for the superposition of their D1 domains with immunoglobulins than with other cell adhesion molecules. In urochordates (*Ciona*) and cephalochordates (*Branchiostoma*), evolutionarily close relatives of the vertebrates, there are homologs of JAM/CTX and nectin IgSF molecules with features of membrane receptors. *Ciona* encodes only a single JAM/CTX-like molecule and two nectin-like molecules [20]. In humans, these molecules are all part of a single linkage group involved in immune function [17],[20]. Taken together, these results suggest that relatively few JAM/CTX and nectin family IgSF molecules were maintained in invertebrates, and the expansion and duplication resulting in the evolution of immunoglobulins may have occurred after the introduction of these molecules into the vertebrate genome.

**Table 2:** Dali Search for JAM-A D1 Structural Homologs.

Hit Number	Z-score^a^	r.m.s.d. (Å)^b^	Percent Identical	Protein	PDB Code-Chain
1–9	24.1–20.3	0.0–0.7	100–65	hJAM-A and mJAM-A	
10	14.6	1.8	16	IgA Fab J539 light chain	2FBJ-L
264	13.0	2.4	17	VCBP3	2FBO-J
543	11.7	2.3	22	Dscam	2V5R-A
572	10.5	2.1	19	NCAM	1IE5-A

a A Z-score above ([number of residues/10]–4) is considered significant.

b r.m.s.d., root mean square deviation.

There also is evidence of expansion of IgSF molecules in invertebrates. For example, like many immunoglobulins, chitin-binding protein (CBP) of *Branchiostoma* is a close structural homolog of JAM-A (Table 2). Variable region-containing (V) CBPs contain a V-type immunoglobulin domain with extensive sequence diversity in the N-terminal region [21],[22]. This diversity is thought to result from high haplotype variation, including variable copy number, polymorphisms, and potential for alternative splicing [23]. Another of the closest structural homologs of JAM-A is Down syndrome cell adhesion molecule (Dscam), an IgSF member of the more evolutionarily distant invertebrate *Drosophila* (Table 2). Dscam is an immune mediator found in clusters of variable exons flanked by constant exons [24],[25]. Thousands of different Dscam molecules can be generated via alternative splicing, a mechanism that is highly conserved across insect orders [26]. Secreted isoforms of Dscam circulating in insect hemolymph contribute to phagocytic uptake of bacteria. While the structural similarities between JAM-A and VCBP or Dscam may not indicate a direct evolutionary relationship, it is clear that diversification and secretion of soluble forms of IgSF molecules can occur in invertebrates and raise the possibility that pathogens have had selective influence on the diversification and secretion of these molecules. Thus, IgSF proteins that served as precursors to soluble adaptive immune effectors may have diversified both prior to and following their introduction into the vertebrate genome. A more thorough examination of IgSF members in invertebrates may clarify mechanisms that led to the evolution of modern adaptive immune mediators and the role of JAM/CTX family molecules in this evolutionary process.

The evolution of JAM family members prior to the biochemical means to efficiently and extensively diversify antigen receptor genes, along with the structural similarities in the binding surfaces of virus receptors and immunoglobulins, provides strong support for the contention that viruses and perhaps other pathogens that engage IgSF receptors contributed to the selection of humoral mediators of adaptive immunity. These observations provide a new framework for understanding how pathogen–host interplay during a prolonged period of evolutionary struggle may have led to the development of antigen-specific immune responses in vertebrates.
